# Correlates of Attitudes toward Sexual Minorities among Vietnamese Social Work Practitioners

**DOI:** 10.3390/ijerph20054241

**Published:** 2023-02-27

**Authors:** Trang Mai Le, Nilan Yu, Stephanie Webb

**Affiliations:** Justice and Society, University of South Australia, Adelaide 5072, Australia

**Keywords:** social work practitioners, Vietnam, attitudes toward sexual minorities, correlates of attitudes

## Abstract

This article examines the correlates of Vietnamese social work practitioners’ attitudes toward individuals who identify as lesbian or gay. This study, among the very few studies on the general topic in non-Western contexts and the first of its kind in Vietnam, investigates correlates of attitudes toward sexual minorities that are known in the literature. The data are drawn from a survey of 292 Vietnamese social work practitioners. The findings suggest that the attitudes of Vietnamese social work practitioners are associated with gender, educational attainment, level of social work education, practice experience, practice sector, professional contact with sexual minority clients, personal contact with sexual minorities, exposure to content on sexual minorities in social work courses and professional development activities, and independent learning activities about sexual minorities but not with age, religious affiliation, and marital status. Implications for social work education and practice are considered.

## 1. Introduction

Prejudice and discrimination can hinder sexual minorities from accessing essential social services [1]. Social workers have a critical role to play in supporting sexual minorities in grappling with such challenges [2,3]. While social workers are more likely to hold positive attitudes toward sexual minorities compared to people outside the helping profession and research has demonstrated a progressive change in attitudes among social work practitioners toward sexual minorities [4,5,6,7,8,9,10,11], it has also been consistently observed over the last few decades that a small but notable proportion of social workers hold negative attitudes toward sexual minorities [2,12,13,14].

As evident in the review of the literature below, social work literature and research have struggled to keep abreast with the expanding scope of the challenges that lie before the profession in terms of the parts of the world where social work is practiced and work with sexual minorities as an area of practice. A majority of what we know about this aspect of practice comes from Western countries, such as the US or Australia. Studies exploring social work practitioners’ attitudes toward sexual minorities in other parts of the world, especially in Asia, are exceedingly rare. As attitudes toward sexual minorities differ across cultures [15], findings from research with Western social work populations cannot be generalised to other non-Western countries such as Vietnam, where social work is an emerging profession. This article reports a component of a larger study—among a few in Asian contexts and the first of its kind in Vietnam—that looked into the attitudes of Vietnamese social work practitioners toward sexual minorities. The findings about the participants’ attitudes have been reported elsewhere (see [1]). The purpose of this article is to discuss the factors related to the attitudes of Vietnamese social work practitioners toward sexual minorities. 

## 2. Review of the Literature

Studies that have attempted to examine the correlates of attitudes toward sexual minorities within social work practice have been few and far between. While there has been a steady flow of research on this topic in Western contexts more broadly, the same cannot be said for research in social work which is the discipline area in which this study was undertaken. For this reason, some of the literature covered in this review is necessarily dated which, in itself, constitutes an argument for studies such as this. The influence of key variables on social workers’ attitudes toward people who identify as lesbian or gay appears to be mixed. While some studies have indicated that older social workers exhibited more negative attitudes toward sexual minorities than younger practitioners [4,11,13], others have found no significant correlation between age and antigay attitudes [10,12,16]. Likewise, religion has been found to be negatively correlated with social workers’ attitudes toward sexual minorities [8,10,12,13,16] although there are notable exceptions (see, for example, [4]). As for other variables such as gender, marital status, and attendance at workshops covering sexual minority content, male social workers have been found to be more heterosexist than female social workers [12], married social workers held more antigay attitudes compared to their never-married-before counterparts [13,16], and training covering sexual minority content can have a significant effect on social workers’ attitudes [4,8]. However, numerous other scholars reported no significant correlation between such variables as gender [4,7,11,16], marital status [4,12], as well as training covering sexual minority content [12,16] and social workers’ attitudes toward sexual minorities. No significant correlation has also been found between such attitudes and income [12] and practitioners’ education level [4,10,11,12]. More consistent findings are reported on such variables as personal contact with sexual minority people and respondents’ sexual orientation. Those who have more personal contact with people who identify as lesbian or gay were reported to hold more favorable attitudes toward sexual minorities [4,8,10,12,13,16], practitioners who served sexual minority clients at work were less likely to hold homophobic attitudes [12,16], and sexual minority social workers tended to have lower levels of homophobia and heterosexism compared to their heterosexual peers [4,13]. Another notable finding is that those employed in public agencies showed more antigay attitudes than those who worked in private settings [13]. This particular finding suggests the possible relevance of the organisational climate, which refers to the shared values, principles, and perceptions of the work environment [17,18,19,20]. Previous studies suggested that the organisational climate could impact workers’ attitudes and their ability to deliver quality services, which would affect the service outcomes and clients’ experiences [17,18,21]. These variables were considered in the study.

## 3. Methodology

### 3.1. Sample and Data Collection

Research packages including the participant information sheets and questionnaires were distributed to social work practitioners working in 14 social centres and social work departments in Hanoi, Vietnam. Completion of the questionnaire required participants to complete a paper and pencil version of the survey. Participants returned the questionnaires by placing them in a locked box within the premises of each organisation. A total of 305 social work practitioners completed the questionnaire. Two participants identified as a sexual minority (*n* = 2) and were excluded from the analysis. An additional 11 cases were removed due to excessive missing data (e.g., data missing on 2 or more key attitude variables). A final sample of 292 participants were included in the quantitative data analysis (see Table 1 for a description of the sample). 

### 3.2. Measures

Attitudes toward sexual minorities were assessed using a translated version of the Attitude Toward Lesbians and Gay Men (ATLG) scale [22]. The ATLG is one of a number of scales developed for measuring attitudes toward sexual minorities. The ATLG was chosen for this study in view of the fact that, unlike other scales, it has been validated in non-Western contexts and also allows for a more granular exploration of any differences in attitudes toward gay men and lesbian women. While the majority of existing scales explore attitudes toward homosexuality in general, the ATLG enables the separate measurement of attitudes toward gay men and lesbians through its two subscales (the ATL and the ATG). It was for these reasons that the ATLG was chosen for the current study. The ATLG has been found to be valid and reliable in assessing attitudes toward sexual minorities among various population samples [23,24,25,26]. The scale has been validated with English-speaking samples [16,27,28,29] and translated versions of the scale have been tested for validity with a number of non-English-speaking samples, including Asian populations [24,30,31] and even Asian samples with social work backgrounds [32]. The scale is in current use in a wide range of fields including sexuality research [33], environmental research and public health [34], psychology [35], homosexuality studies [36], pedagogy [37], psychiatric care in nursing [38], and sports [39]. The ATLG consists of 20 different statements, 10 about gay men (Attitude Toward Gay Men—ATG subscale) and 10 about lesbian women (Attitude Toward Lesbians—ATL subscale) to which respondents indicate their levels of agreement or disagreement (Herek, 1994). To avoid midpoint tendency among the targeted sample, the ATLG scale was administered using 6-point response options with 1 being strongly disagree and 6 being strongly agree. With 6-point Likert responses, the total ATLG score could range from 20 to 120, and ATL and ATG scores could range from 10 to 60. Higher scores indicate more negative or unfavorable attitudes toward sexual minorities. For the current study, the ATLG, the ATL, and the ATG all demonstrated excellent internal reliability (αATLG = 0.95; αATL = 0.91; αATG = 0.91).

Several demographic variables were collected. Age was reported in years. Gender identity was recorded categorically as female and male. Marital status was dichotomously recorded as ‘single/never married’ and ‘married/previously married’. Religion was reported as non-religious, Buddhism, Christianity, and other. Highest education attainment was reported as vocational training, college degree, bachelor’s degree, master’s degree, and doctorate degree. Given the research context in Vietnam, where social work practitioners can come from different backgrounds and their social work education may not necessarily be their highest educational attainment, the survey included a separate question regarding the participants’ social work education. The participants were asked to indicate the highest level of social work education that they obtained with choices of ‘none’, ‘training/short courses’, ‘college degree’, ‘bachelor’s degree’, ‘master’s degree’, and ‘doctorate degree’. Practice sector was recorded as a dichotomous variable (public sector/governmental organisations and private sector/non-governmental organisations). Professional contact with sexual minorities, personal contact with sexual minorities, and education about sexual minorities were assessed by dichotomous (no/yes) survey questions. 

The survey instrument was translated into Vietnamese following a back-translation technique for cross-cultural research [40]. The English version of the questionnaire was translated into Vietnamese by the researcher who is bilingual in English and Vietnamese. The translated version was then blindly translated back into English by two independent Vietnamese social work researchers who were proficient in English and had never seen the original questionnaire. A comparison was made and changes were introduced as needed to correct any mistranslation and to ensure consistency. 

### 3.3. Data Analysis

The survey data were analysed using the Statistical Package for Social Sciences software (SPSS version 25). Univariate analysis was employed to provide descriptive information on the sample and the participants’ attitudes toward sexual minorities. Reliability analysis was run to report the Cronbach’s coefficient alphas of the ATLG for the sample of Vietnamese social work practitioners. One-way ANOVA, independent samples *t*-tests, and bivariate correlations were undertaken to examine the relationships between all variables and to determine which predictors would be included in the multiple regression analysis in step 2.

### 3.4. Ethics

Ethics approval for this project was granted by the Human Research Ethics Committee of the University of South Australia (Ethics Protocol 201010).

## 4. Results

### 4.1. Preliminary Analysis

Bivariate correlations and independent samples *t*-tests were used as preliminary analysis for the key predictors. Overall, participants who obtained higher levels of education or higher social work education, and with more years in practice, held more positive attitudes toward sexual minorities. Results indicated that age was, on the whole, not a factor associated with participants’ attitudes toward sexual minorities. However, when attitudes were broken down between gay men and lesbian women, a significant correlation was found between age and attitudes toward lesbian women, suggesting that older participants held more negative attitudes toward people who identified as lesbian (see Table 2 for correlations of all study variables). Practitioners who served in the public sector (*M* = 58.02, *SD* = 13.73) held significantly more negative attitudes toward sexual minorities than those in the private sector (*M* = 44.34, *SD* = 12.82), *t*(290) = 7.19, *p* < 0.001. Those who reported knowing sexual minorities (*M* = 40.46, *SD* = 8.91) were less prejudiced than those who did not have such contact (*M* = 58.42, *SD* = 13.62), *t*(123.94) = 12.09, *p* < 0.001. Practitioners who reported having experience working with sexual minorities/professional contact with sexual minorities (*M* = 40.50, *SD* = 11.46) held significantly more positive attitudes than those who had no such experience/such contacts (*M* = 56.63, *SD* = 14.08), *t*(290) = 6.05, *p* < 0.001. Those who reported having taken academic courses covering sexual minority content (*M* = 39.97, *SD* = 6.98) were significantly less prejudiced than those who did not (*M* = 56.88, *SD* = 14.28), *t*(72.62) = 11.24, *p* < 0.001. Results also indicated that participants who had professional training covering sexual minority content (*M* = 39.67, *SD* = 9.09) had significantly less negative attitudes than those who did not have such training (*M* = 58.21, *SD* = 13.53), *t*(103.11) = 12.02, *p* < 0.001. Likewise, those who engaged in independent learning (*M* = 43.17, *SD* = 8.23) held more positive attitudes than those who did not (*M* = 62.75, *SD* = 12.64), *t*(289.97) = 16.03, *p* < 0.001. Furthermore, an independent samples *t*-test showed that male practitioners (*M* = 62.55, *SD* = 13.51) were more prejudiced toward sexual minorities than were female practitioners (*M* = 50.96, *SD* = 13.66), *t*(290) = 6.93, *p* < 0.001.

There was no significant difference in ATLG mean scores between those who were never married (*M* = 53.38, *SD* = 14.57) and those who were married or previously married (*M* = 55.47, *SD* = 14.61), *t*(288) = −1.08, *p* = 0.282. No statistically significant differences in attitudes were found between religious affiliations, *F*(2, 288) = 0.75, *p* = 0.475.

### 4.2. Multivariate Analysis

Two multiple regression analyses were conducted to assess which variables predict attitudes toward gay men and attitudes toward lesbian women separately. Preliminary analyses were conducted to ensure no violation of the assumptions of normality, linearity, multicollinearity, and homoscedasticity. All assumptions were met.

#### 4.2.1. Attitudes toward Lesbian Women

Age, gender, education level, social work education, years of practice, practice sector, personal contact, professional contact, academic courses covering sexual minority content, professional training covering sexual minority content, and independent learning activities about sexual minorities, when entered into the regression, collectively explained 55.4% of the variance in the attitudes toward lesbian women (see Table 3 for model summary of ATL multiple regression). In the final model, only age, education level, social work education level, years of practice, personal contact with sexual minorities, professional training covering sexual minority content, and independent learning about sexual minorities remained statistically significant in predicting attitudes toward lesbian women, *F*(11, 277) = 33.51, *p* < 0.001. Age had the highest beta value (*β* = 0.14, *p* < 0.05).

#### 4.2.2. Attitudes toward Gay Men

A second, separate multiple regression was run to predict attitudes toward gay men. The aforementioned variables, excluding age, explained 62.5% of the variance in the data, F(10, 281) = 49.41, *p* < 0.001. However, in the final model, only gender, education level, social work education level, professional training covering sexual minority content, and independent learning about sexual minorities remained statistically significant (see Table 3 for model summary of ATG multiple regression). Professional training covering sexual minority content had the highest beta value (β = −0.115, *p* < 0.05).

## 5. Discussion

The survey findings suggest that the attitudes of social work practitioners were associated with certain sociodemographic variables such as gender, educational attainment, social work education, social work experience, practice sector, professional contact with sexual minority clients, personal contact with sexual minorities, exposure to content on sexual minorities in social work courses and professional development activities, and independent learning activities about sexual minorities. No association was found between the participants’ attitudes toward sexual minorities and their age, religious affiliation, and marital status.

The findings suggest that there are some significant factors differentially associated with attitudes toward lesbian women as against attitudes toward gay men. While lower levels of education, less social work education, fewer opportunities to obtain professional training covering sexual minorities, and less independent learning about sexual minorities were associated in common with negative attitudes toward lesbian women and negative attitudes toward gay men, there were factors specifically associated with one or the other. Older people and those with fewer years of experience and less contact with sexual minorities were more likely to hold negative attitudes toward lesbian women. It could be traditional ways of thinking that older people tend to subscribe to and fear of the unknown Other that comes with less work experience and contact with sexual minorities that underpin such attitudes. Unlike the attitudes toward lesbian women, however, age, professional experience, and contact with sexual minorities did not figure in attitudes toward gay men. Perhaps, most notably, males were more likely to hold negative attitudes toward gay men.

On the whole, female social work practitioners were found to hold more positive attitudes toward sexual minorities than their male counterparts. This is consistent with findings from previous studies carried out with members of the general public (see, for example, [31,41,42,43,44]). Gender differences in attitudes toward sexual minorities have been widely investigated [16,45]. Evidence suggests that heterosexual males express more negative attitudes toward people who identify as lesbian or gay than heterosexual females [31,41,42,43,44]. In addition, while heterosexual females’ attitudes toward lesbian women and gay men do not markedly differ, heterosexual males tend to hold more negative attitudes toward men who identify as gay than toward women who identify as lesbian [26,42,43,44,46,47,48,49]. This phenomenon could be explained by the fact that the expected male role in almost every culture is attached to masculinity. It has been posited that males may feel pressured to affirm and project their masculinity by performatively demonstrating overt and outright disapproval toward those who go against this cultural expectation [42,50]. Not doing so puts them at risk of being labelled homosexual or, at the very least, being sympathetic to and guilty by association with sexual minorities. Holding negative attitudes toward sexual minorities can thus be a way through which men confirm their masculinity and maintain their privileged status as bona fide heterosexual males [43]. In contrast, heterosexual women are not burdened with parallel social pressure to reaffirm their feminine identity through antigay bias and negative attitudes. Even when displaying traits traditionally associated with masculinity, women are less likely to be thought of as being lesbian [51]. Thus, while men and women may potentially feel the same level of discomfort toward sexual minorities, heterosexual men tend to display more antigay aggression compared to heterosexual women [43] (Herek & McLemore, 2013).

The finding of a significant correlation between gender and attitudes toward sexual minorities, however, contrasts with most of the studies conducted with social work practitioners [4,7,11,16]. This may have to do with the gender profile of the study participants. As noted by Chonody and Smith [5], studies with social workers tend to have an overwhelmingly greater representation of female participants in the sample due to the overrepresentation of female practitioners in the field. As such, it could limit the determination of attitudinal differences based on gender, if such differences exist [5]. Similarly, Zamora [11] argued that, of studies which found no correlation between attitudes and gender, male social workers were often underrepresented in the sample. Unlike the present study, most past studies have notable disparities in the gender representation, with female participants representing 80% or more of the sample. The current study has relatively higher representation of male participants, with men constituting 34.6% of the sample. This may account for the different finding regarding gender and attitudes compared with previous social work research.

The results of this study indicated a significant association between attitudes toward sexual minorities and educational attainment as well as the level of their social work education. In most previous studies conducted with social work practitioners, the variable ‘educational attainment’ is often equated with formal training in social work. This is not the case for the current study, given the research context in Vietnam where social work practitioners may come from various educational backgrounds and any formal social work education experience may not necessarily be their highest educational attainment. For this reason, the participants’ educational levels were explored through two variables: ‘educational attainment’ and ‘social work education attainment’. The study found a positive correlation between educational attainment and accepting attitudes toward sexual minorities. Participants with higher levels of education tended to hold more positive attitudes toward sexual minorities. This finding is in line with findings from a number of studies involving members of the general public, such as those of Herek and McLemore (2013) [43], Yu et al., (2011) [31], Lewis (2003) [4], and Kelley et al., (2001) [40]. However, the finding that practitioners with higher social work education espoused more positive attitudes toward sexual minorities is not consistent with what Berkman and Zinberg [12], Crisp [16], Bossenmeyer [4], and Zamora [11] reported. It should be noted, however, that the majority of these studies had samples largely consisting of social workers with Master of Social Work (MSW) degrees; for example, MSW participants represented 93% of the sample in Crisp’s [16] study and 83.5% of Bossenmeyer’s [4] study. It may be problematic when attempting to identify the attitudinal differences by social work education level when the sample is predominantly made up of participants holding a particular type of social work degree. The study of Zamora [11] had a more diverse sample, with participants holding not only Bachelor of Social Work (BSW) and Master of Social Work (MSW) degrees, but also bachelor’s degrees and master’s degrees in other disciplines. Zamora [11] found social workers holding an MSW had more positive attitudes toward lesbians than those holding a BSW. Although this was not found to be significant, the near significance did suggest the need for further research on the role of social work education in shaping attitudes [11]. The sample of the current study had more marked variations in social work education attainment, with participants obtaining varied social work educational qualifications, from vocational certificates and college diplomas, to bachelor’s degrees or master’s degrees (see Table 1). The study shows that the higher the participants’ social work education levels, the less bias they felt toward sexual minorities. Tolar and associates [52] and Swank and Raiz [53] suggested that social work education—which generally embraces compassion, professional values, social justice, human rights, and inclusion—may have liberalising effects and lead to more open-minded and non-judgmental individuals.

The finding of a significant correlation between years of professional experience and attitudes toward sexual minorities contrasts with previous studies that have explored this relationship among social workers [4,16]. For example, Crisp [16] had found that time spent in direct practice has no association with attitudes toward sexual minorities. The results in this study, on the contrary, suggest that the more experienced social work practitioners are in direct practice, the more likely it is that they hold positive attitudes toward sexual minorities. It is possible that practicing social work has had a positive influence on the participants’ attitudes toward those who identify as lesbian or gay. How this influence occurs is open to question. Swank and Raiz [53] (p. 63) contended that the very act of doing social work ‘can generate greater levels of empathy, respect, and admiration for GLBT individuals’. They surmised that the introduction to the profession can foster a greater awareness of the inequities sexual minorities face, leading to the enhancement of empathy toward them [53]. In addition, there is the possibility that the more time participants engaged in direct practice, the more likely it is for them to have accessed in-service training and professional development activities. Such educational experiences could potentially provide exposure to values and ways of thinking that engender more accepting attitudes, as discussed above. This may explain the more positive views among practitioners who had more years of professional experience in this study. However, all these are put into question upon closer inspection of the findings. While the amount of practice experience is associated with attitudes toward lesbian women and with attitudes toward sexual minorities in general, the same cannot be said in relation to attitudes toward gay men. So, the practice of social work and the potential access to professional development opportunities that this may bring appear to have no bearing on the practitioners’ attitudes toward gay men. This may have to do with the standard of male masculinity discussed earlier.

The practice setting—that is, whether the participants worked in a governmental/public sector or non-governmental/private sector organisation—was found to be associated with participants’ attitudes toward sexual minorities in general. This study found that participants working in the private sector/non-governmental organisations held more positive attitudes than practitioners in public sector/governmental organisations. It is perhaps understandable that this happens in countries such as Vietnam, where administrators and workers in public sector/governmental organisations are expected to strictly abide by and not go beyond official pronouncements and positions. Public sector organisations are widely perceived in Vietnamese society as being more conservative compared to private sector organisations. However, such differences between practice settings have also been observed elsewhere. In their study of mental health professionals in the US, the most proximate of its kind, albeit dated, De Crescenzo and McGill [13] found that those who worked in public agencies tended to see gay people as sick more than those who worked in private agencies. It is noteworthy that this variable was not consistently explored in previous studies of social workers. To the knowledge of the researchers, except for the study of De Crescenzo and McGill [13], there is no recent study which included information on social workers’ practice setting nor explored the relationship between this variable and practitioners’ attitudes toward sexual minorities. It may be that the practice setting has not been seen by other researchers, mainly from Western contexts, as playing an important role in attitudes toward sexual minorities compared with other variables. This appears not to be the case in the current study. The differences in the organisational culture between public sector and private sector organisations in Vietnam may account for the differences in practitioner attitudes between practice settings. Another possible explanation suggested by the data is that those who worked in private sector organisations were more likely than those who worked in the public sector to receive professional training about sexual minorities. As noted earlier, educational experiences about sexual minorities may have a positive effect on practitioners’ attitudes.

The results of this study also reveal that those who engaged in practice with sexual minorities held more positive attitudes than those who worked in other practice fields. It may be the case that the social workers who were working in this area of practice were there precisely because they held positive attitudes toward sexual minorities and were thus inclined to engage in such work. Swank and Raiz [53] suggested that organisations that catered to sexual minorities tended to recruit and retain practitioners who are open to sexual diversity. Therefore, practitioners who work in this field of practice tend to be more open-minded and less judgmental toward lesbian or gay persons. However, it is possible that working in such an area could influence practitioner attitudes in at least two ways. Such work would orient practitioners to knowledge and values that foster acceptance and tolerance toward sexual minorities. It was discussed earlier how such a learning process could occur. Moreover, it could also be that the experience of working directly with sexual minorities increased the participants’ interaction with these populations. As noted earlier and supported by previous studies [12,16], such professional contact appears to promote understanding of sexual minorities, reduce ignorance, and minimise prejudicial attitudes. In interpreting the attitudinal differences based on engagement with particular areas of practice, however, it is important to note that in the current study, the sample size of practitioners working with sexual minorities was extremely small. The research was not designed to explore this question in depth. Thus, this finding must be regarded as tentative, at best. Future research with an expanded sample size to cover the range of practice fields will be needed to verify whether such association does exist and, if so, what the nature of this association is.

The finding that contact with sexual minorities was associated with attitudes toward them is in line with findings from past studies involving members of the general public [26,44,54]. Both personal and professional contact with sexual minorities was found to be significantly correlated with participants’ attitudes, suggesting that contact with sexual minorities, whether they are acquaintances (friends, family members, colleagues) or clients, in some way is linked to practitioners’ attitudes toward these populations. Respondents with sexual minority acquaintances held significantly more positive attitudes toward sexual minorities than those who did not have such contact. Perhaps more telling, similar results have also been found in previous studies with social workers [4,8,10,12,13,16]. Practitioners who had professional contact/practice experience with sexual minority clients had significantly lower levels of prejudicial attitudes toward these populations than those who did not. Crisp [16] and Berkman and Zinberg [12] indicated that practitioners who served sexual minority clients at work were less likely to hold homophobic attitudes. This result supports the position taken by Pettigrew and Tropp [55], who argued that intergroup contact promotes attitude changes because such contact is likely to improve knowledge about the outgroup, reduce anxiety, and increase empathy toward these populations. However, as with gender above, there was a difference at the granular level. While contact with sexual minorities was associated with attitudes toward lesbian women and with attitudes toward sexual minorities in general, it is not a factor predicting attitudes toward gay men. It is unclear what accounts for this apart from that entrenched view of male identity discussed above.

The finding that education and training covering sexual minority content was correlated with social work practitioners’ attitudes toward sexual minorities is in line with previous findings reported by Bossenmeyer [4] and Krieglstein [8] but differs from those of Berkman and Zinberg [12] and Crisp [16]. The findings in the literature are thus inconclusive, with issues possibly stemming from notable variations in approaches to education and training about sexual minorities. In an attempt to gain a better grasp of this variable, the current study incorporated a number of questions to explore different types of training, including formal academic courses, professional development training, and independent learning activities. The results revealed significant correlations between attitudes toward sexual minorities and all these three forms of education, suggesting that a wide range of learning experiences can engender positive attitudes toward these populations. Professional training and independent learning covering sexual minority content, in particular, were strongly associated with attitudes toward lesbian women and gay men. For those who engaged in independent learning activities, it may be that such participants already had positive attitudes to begin with, which in turn prompted them to proactively engage in self-directed learning and seek information about sexual minorities. However, it appears that education and training covering sexual minority content may have had a positive effect on the attitudes of the participants. More research would be needed to explore this.

The finding of no significant relationship between marital status and social work practitioners’ attitudes toward sexual minorities is consistent with what was reported by Berkman and Zinberg [12] and Bossenmeyer [4] but different from the findings of De Crescenzo and McGill [13] and Crisp [16]. Again, it could be said that the literature is inconclusive in this respect. Contrary to popular belief and the results of a number of studies conducted with general populations, the finding in this study in relation to age suggests no association with the participants’ attitudes toward sexual minorities. However, the finding of no association between age and attitudes toward those who identify as lesbian or gay is consistent with previous studies among social work practitioners [10,12,16]. Although there was no association between age and attitudes toward sexual minorities as a whole, a significant correlation was found between age and attitudes toward lesbian women, suggesting that older participants held more negative attitudes toward people who identified as lesbian. It is worth noting, however, that the age range of the participants (to wit: 23 to 53) was not considerably wide, which tempers this finding.

In contrast with what has been largely reported in the literature (see, for example, [8,10,12,13,16]), there were no substantial differences in attitudes on account of religious affiliation, suggesting that religiosity was not a factor associated with social work practitioners’ attitudes toward sexual minorities. A possible explanation as to why the present study found different results is the different sociocultural contexts of the research. While the majority of the abovementioned studies were conducted in Western contexts where ‘religious’ participants may be mainly adherents of different variants of Christianity, the present study was conducted in an Asian country with a predominantly non-Christian population [56]. It is also possible that the small sample size (14.7%) of participants who self-identified as having religious affiliations did not allow the detection of any differences. Moreover, it could be that religious affiliation was not adequately measured in the current study’s data gathering instrument. There is also the question of what constitutes a religion and religious identity. Vietnamese people may not consciously identify as adherents of a particular religion, but they may be unconsciously bound up with religious/spiritual beliefs and outlooks so preponderant as to be rendered invisible [56,57,58]. Thus, examining the relationship between religiosity and attitudes toward sexual minorities may require more effort than just the two questions asked in the questionnaire about participants’ religious affiliations which were patterned after approaches of similar research undertaken in Western contexts.

### Limitations

The study has several limitations that are worth noting and taking into consideration. First, this study was conducted with social work practitioners in a number of Hanoi-based organisations. The selection of social work organisations was limited to those from whom institutional permission could be obtained. The use of such a non-probability sample in the study limits its generalisability. The findings cannot be generalised to social work practice across Vietnam, much less beyond Vietnam.

Another limitation relates to the sampling design. Due to the voluntary nature of the study, the participants in the survey were self-selected. Despite the high response rate, it is possible that social work practitioners who responded to the survey may have had a particular disposition, perhaps a more-than-average interest in sexual minority issues, while those who had negative attitudes tended to opt out of the study. The self-selection process could have resulted in a highly skewed sample.

Finally, as noted in the discussion, the study could have been designed better to adequately measure participants’ religiosity in the questionnaires. Measuring religiosity in a country such as Vietnam where people may self-identify as non-religious while unconsciously immersed in particular religious/spiritual worldviews is a challenge in and of itself. Exploring the potent influence of religiosity on attitudes toward sexual minorities in a country such as Vietnam requires more effort than the one or two questions on religious affiliation that are often found in mainly Western standardised instruments. A measurement of religiosity specifically designed for Vietnamese respondents should be considered and may have to be developed for future research.

## 6. Conclusions

This current study that examined the correlates of attitudes toward sexual minorities among a sample of Vietnamese social work practitioners is a contribution to what has largely been an underinvestigated knowledge area in the discipline where the mostly dated literature provides conflicting evidence. There may be some specificities in non-Western and Asian contexts particularly that can shed light on practice knowledge that has, hitherto, largely been shaped by a Western lens. This underscores the importance of more work in this area and in other parts of the world. Insights into the factors associated with such attitudes have important implications for social work education and practice. Given the findings, there is argument for the provision of education programs focusing sexual minorities along with opportunities for encounters with members of these populations. The findings also suggest that social work education itself in the form of formal education on-the-job professional training and individual learning can contribute to more acceptance of and empathy towards sexual minorities among social work practitioners. This is likely salient in practice settings such as Vietnam where social work practitioners come from a wide range of training backgrounds including those who do not have formal social work education. There are factors that are beyond the immediate control of social work practitioners such as the culture of the organisations that they are employed in and the kind of training provided by their employing organisations. However, there are factors over which they have direct influence, such as their level of overall education and social work education, personal contact with sexual minorities, and independent learning on sexual minority concerns. Social work practitioners aiming to provide better and more responsive care to sexual minority clients can proactively work towards engendering in themselves these attributes that are associated with positive attitudes toward sexual minorities. This needs to be seen in the context of much broader efforts in building social work as a profession in Vietnam and in other parts of the world where social work is still a nascent profession.

## Figures and Tables

**Table 1:** Demographic information of participants (*n* = 292).

Variable		Frequency	Percentage (%)
Age	20–29	106	36.3
	30–39	127	43.5
	40–49	54	18.5
	Over 50	2	0.7
Gender	Male	101	34.6
	Female	191	65.4
Religion	None	248	84.9
	Buddhism	35	12.0
	Christianity	8	2.7
Marital status	Single/Never married	78	26.7
	Married/Previously married	212	72.5
Education level ^1^	Vocational training	57	19.5
	College	59	20.2
	Bachelor’s	143	49.0
	Master’s	33	11.3
Social work education level	None	18	6.2
	Training/short courses	151	51.7
	College	31	10.6
	Bachelor’s	69	23.6
	Master’s	23	7.9
Years of practice	Under 1 year	31	10.6
	1–3 years	80	27.4
	Over 3 to 5 years	67	22.9
	Over 5 to 10 years	60	20.5
	Over 10 years	54	18.5
Practice sector	Public	227	77.7
	Private	65	22.3

Note: Total percentage of each category might not be 100% due to the missing data. ^1^ In the Vietnamese education system, students enter into vocational training programs after completing the upper secondary education (high school). Those who graduate from (professional) vocational training programs are awarded with vocational training diplomas/certificates. At the higher education level, there are college, undergraduate, master’s, and doctorate programs.

**Table 2:** Bivariate correlations matrix.

	Age	Gender	Education Level	SW Education	Years of Practice	Practice Sector	Professional Contact	Personal Contact	Academic Courses	Professional Training	Independent Learning	ATL	ATG	ATLG
Age	__	0.075	0.004	−0.070	0.656 **	−0.211 **	0.031	−0.198 **	−0.040	−0.078	−0.065	0.116 *	0.093	0.109
Gender		__	0.190 **	0.092	0.175 **	0.112	0.080	0.171 **	0.123 *	0.183 **	0.281 **	−0.290 **	−0.442 **	−0.389 **
Education level			__	0.471 **	0.233 **	0.341 **	0.241 **	0.368 **	0.270 **	0.328 **	0.437 **	−0.524 **	−0.534 **	−0.555 **
SW education				__	0.240 **	0.204 **	0.145 *	0.254 **	0.418 **	0.281 **	0.347 **	−0.542 **	−0.528 **	−0.560 **
Years of practice					__	−0.040	0.134 *	−0.068	0.151 **	0.125 *	0.196 **	−0.243 **	−0.273 **	−0.272 **
Practice sector						__	0.307 **	0.534 **	0.147 *	0.621 **	0.339 **	−0.374 **	−0.374 **	−0.390 **
Professional contact							__	0.380 **	0.057	0.349 **	0.348 **	−0.338 **	−0.290 **	−0.319 **
Personal contact								__	0.156 **	0.463 **	0.493 **	−0.502 **	−0.466 **	−0.495 **
Academic courses									__	0.292 **	0.219 **	−0.394 **	−0.358 **	−0.388 **
Professional training										__	0.364 **	−0.488 **	−0.475 **	−0.500 **
Independent learning											__	−0.664 **	−0.649 **	−0.677 **
ATL												__	0.825 **	0.950 **
ATG													__	0.961 **

* *p* < 0.05. ** *p* < 0.001. All two-tailed tests.

**Table 3:** Results of the Multiple Regression (*n* = 292).

Variable	ATL			ATG		
	B	SE B	*β*	B	SE B	*β*
Age	0.147	0.057	0.140 *	__	__	__
Gender	−1.008	0.628	−0.067	−4.338	0.652	−0.254 **
Education level	−0.964	0.384	−0.125 *	−1.209	0.400	−0.138 *
Social work education level	−1.216	0.325	−0.191 **	−1.593	0.333	−0.219 **
Year of practice	−0.889	0.318	−0.159 *	−0.477	0.252	−0.075
Practice sector	0.140	0.940	0.008	−0.426	0.976	−0.022
Professional contact with sexual minorities	−1.682	1.047	−0.072	−0.556	1.094	−0.021
Personal contact with sexual minorities	−1.944	0.964	−0.108 *	−1.543	1.007	−0.075
Academic courses covering sexual minority content	−1.954	1.004	−0.087	−1.379	1.048	−0.054
Professional training covering sexual minority content	−2.123	1.020	−0.113 *	−2.463	1.066	−0.115 *
Independent learning about sexual minorities	−4.301	0.739	−0.295 **	−5.068	0.766	−0.305 **
*R^2^*	0.571			0.637		
*F*	33.514			49.408		

* *p* < 0.05. ** *p* < 0.001. All two-tailed tests.

## Data Availability

The data presented in this study are available on request from the corresponding author. The data are not publicly available due to privacy and ethical considerations.
